# Validation of Compressed Sensing Accelerated Magnetization Prepared Rapid Acquisition Gradient Echo for Fast and Reliable Brain Volume Measurement

**DOI:** 10.3390/tomography12090121

**Published:** 2026-08-24

**Authors:** Jung Bin Lee, Hye Shin Ahn, Leehi Joo, Younghee Yim

**Affiliations:** 1 Department of Radiology, Chung-Ang University Hospital, Chung-Ang University College of Medicine, Seoul 06973, Republic of Korea; 2 Biomedical Research Institute, Chung-Ang University Hospital, Seoul 06973, Republic of Korea

**Keywords:** brain volume measurement, magnetization-prepared rapid acquisition gradient echo, compressed sensing

## Abstract

**Objectives**: To assess the clinical viability of compressed sensing magnetization, we prepared a rapid acquisition gradient echo (CS-MPRAGE) for brain volume measurement by comparing its scan time and image quality with standard MPRAGE. **Methods**: In this retrospective study, we analyzed a total of 40 morphologically normal MRIs from relatively young subjects (mean age 27.03 ± 5.02, range 19~40, F:M = 33:7) who underwent both CS-MPRAGE and standard MPRAGE. MRI was performed with a 3T MR scanner using a 64-channel head coil. Volumetric data analysis was conducted using commercial AI-powered quantification software. The signal-to-noise ratio (SNR) and contrast-to-noise ratio (CNR_gray matter/white matter_) were measured and compared. Qualitative analysis based on overall image quality, deep gray matter delineation, artifacts, and gray–white matter differentiation was rated on a five-point visual scoring system. Statistical analyses included paired t-tests, intraclass correlation coefficients (ICCs) and Pearson correlation. **Results**: CS-MPRAGE had a significantly shorter scan time than standard MPRAGE (1:46 vs. 5:20 min; *p* < 0.001). Whole-brain volume was similar (standard-MPRAGE, 1176.89 ± 105.36; CS-MPRAGE, 1181.14 ± 105.62 mL; *r* = 0.988; ICC, 0.9754–0.9931). Gray matter and regional lobe volumes were comparable, while the white matter volume was slightly larger with CS-MPRAGE. CNR of the gray–white matter was significantly higher with CS-MPRAGE (left, 30.45 ± 11.21 vs. 49.53 ± 32.71; right, 30.73 ± 11.97 vs. 46.89 ± 30.40; *p* = 0.0001) while SNR were similar across key brain regions. **Conclusions**: CS-MPRAGE provides high-quality 3D images and reliable volume data with significantly reduced acquisition time and comparable image quality.

## Simple Summary

Accurate brain volume measurement is essential for diagnosing and monitoring neurodegenerative diseases, but standard MRI requires long scan times, increasing patient discomfort and motion-related artifacts. Many fast sequences are available, but sufficient validation is lacking, particularly in young, healthy populations. Fast MRI using compressed sensing magnetization-prepared rapid acquisition gradient echo (CS-MPRAGE) provides reliable brain volumetric measurements comparable to standard imaging, while significantly reducing scan time. CS–MPRAGE enables efficient, high-quality brain imaging without compromising the reliability of quantitative brain volumetric measurements.

## 1. Introduction

Recent advances in high-resolution magnetic resonance imaging (MRI), such as three-dimensional (3D) T1-weighted Magnetization Prepared Rapid Acquisition Gradient Echo (MPRAGE), have enabled more detailed assessment of brain morphology and quantitative measurements by providing excellent spatial resolution and tissue contrast. In parallel, advances in artificial intelligence now facilitate fully automated whole-brain segmentation and volume measurements, which are increasingly applied in clinical practice [1].

In neurodegenerative disease, quantitative volume measurement is essential for diagnosis, disease monitoring, and therapeutic evaluation, as visible structural changes often remain subtle in early disease stages [2]. Whole-brain volume measurement can be done using high-resolution three-dimensional T1 MPRAGE [3]. However, the relatively long acquisition time, which is required to encode a large number of k-space lines, the preceding inversion time (TI), and the necessity of T1-weighted contrast [4], poses practical challenges. Extended scan duration increases patient discomfort and susceptibility to motion artifact, which can degrade image quality and reduce measurement accuracy.

To overcome the hurdles, several acceleration strategies have been proposed to reduce scan times, including compressed sensing (CS), generalized autocalibration, partially parallel acquisitions (GRAPPA), or wave-controlled aliasing in parallel imaging (CAIPI) [4,5,6]. CS achieves image acquisition acceleration by applying sparsity constraints and k-space undersampling [7]. In recent years, the application of CS acceleration to routine brain MRI has demonstrated substantial reductions in acquisition time without significant loss of image quality [8]. Recently, a study applied ultrafast MPRAGE employing wave-CAIPI as acquisition acceleration have shown comparable morphometric estimates and low intra-individual variability relative to the standard protocol [9]. Nonetheless, evidence regarding the consistency and quantitative validity of volumetric measurements obtained using CS-accelerated MPRAGE remains limited in clinical settings.

To address this gap, the present investigation aimed to evaluate the agreement between volumetric measurements obtained with and without CS acceleration. By systematically comparing quantitative outputs from both sequences, this study sought to determine whether CS-accelerated MPRAGE provides reliable volumetric data suitable for clinical application. Our findings have clarified the potential of CS-accelerated MPRAGE as an alternative to conventional MPRAGE in quantitative neuroimaging workflow, potentially enhancing imaging efficiency while maintaining the reliability of volumetric measurements.

## 2. Materials and Methods

This study compared the brain volumetric measurements and image quality between standard MPRAGE and CS-MPRAGE. The methods and results have been reported in accordance with the Standards for Reporting of Diagnostic Accuracy Studies (STARD) guidelines [10]. All methods were approved by the Chung-Ang University Hospital Institutional Review Board (IRB) No. 2502-004-19561. The Institutional Review Board approved this retrospective study and waived the requirement for informed consent.

### 2.1. Study Population

**Figure 1:**
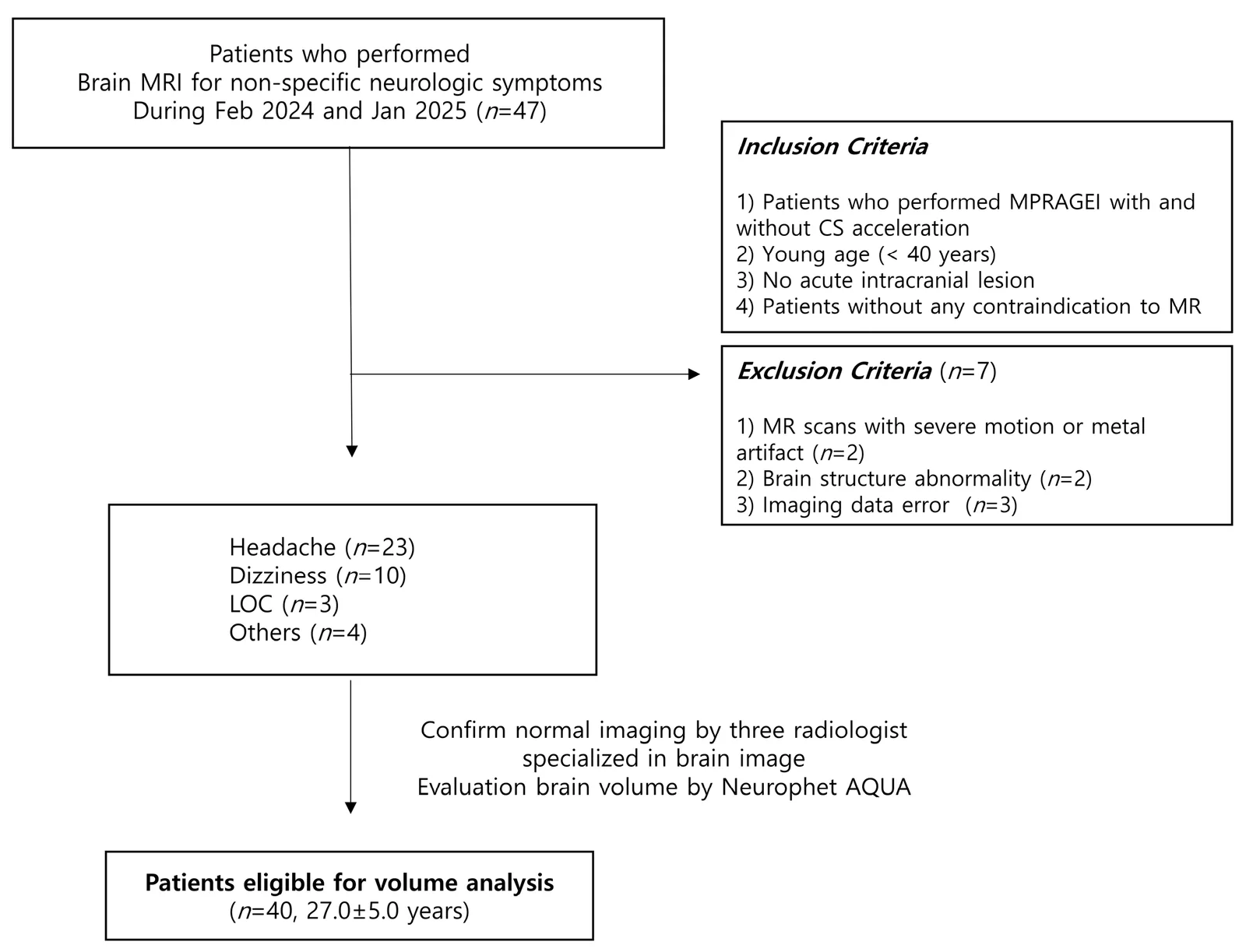
Study flow chart.

This retrospective study included 47 consecutive patients who underwent brain MRI for nonspecific neurologic symptoms, such as headache and dizziness, between February 2024 and January 2025 (Figure 1). Inclusion criteria were as follows: (1) patients aged 19–40 years who underwent both standard and CS-MPRAGE sequences during the same imaging session, (2) absence of acute intracranial lesion and (3) no contraindication to MR. Exclusion criteria were as follows: (1) scans with severe motion or metal artifacts, (2) presence of brain abnormalities such as infarction, hemorrhage, or tumor, and (3) corrupted or incomplete imaging data. Demographic and clinical data including age, sex and final diagnosis were obtained from electronic medical records (EMR). By reviewing the EMR, three radiologists, each with over 10 years of experience determined and confirmed that there were no organic problem which can hamper volume measurement by comprehensively evaluating all available clinical information, patient progress, and imaging data.

### 2.2. Image Acquisition and Reconstruction

All MRI examinations were performed on a clinical 3 T scanner (MAGNETOM Vida, Siemens Healthcare, Erlangen, Germany) using a 64-channel head and neck array coil with the patient in the supine position. Detailed acquisition parameters for both standard and CS–MPRAGE sequences are summarized in Table 1. The prototype CS–MPRAGE reconstruction algorithm was provided by the manufacturer (Siemens Healthcare, Erlangen, Germany) for clinical evaluation.

### 2.3. Quantitative Analysis

Both standard and CS-MPRAGE datasets were processed using automated segmentation software (Neurophet AQUA, AQ-3.0.2) [11,12]. Each dataset was uploaded to the server of the software tool in Digital Imaging and Communications in Medicine (DICOM) format for volumetric analysis. All segmentation results were visually inspected. Cases with unsuccessful automated segmentation or volumetric measurement were excluded from the analysis. No manual correction of the automated segmentation results was performed.

A radiologist with 11 years of experience performed the quantitative analysis and calculated the signal-to-noise ratio (SNR) based on the signal intensity (SI) of the bilateral centrum semiovale, putamen, cerebellum and pons using a manually drawn circular region of interest (ROI) on axial images. All ROIs (approximately 0.5 cm^2^) were placed in the homogeneous brain parenchyma. The standard deviations (SDs) of SI were also recorded within each ROI. Due to the non-homogeneous noise distribution of the parallel acceleration images, we avoided direct measurement of the noise from the background and obtained the value from the SD of the white matter instead, as described previously [13,14,15]. To increase the reliability of the extracted values, the rater measured the SI three times, and the mean value was used for the analysis.

### 2.4. Qualitative Analysis

Two board-certified radiologists independently reviewed all MPRAGE images in random order using a commercial picture archiving and communication system (PACS). Both readers were blinded to sequence type, clinical information, and original radiological reports. Each image set was evaluated for overall image quality, gray–white matter differentiation, and deep gray matter delineation, rated on a five-point scale: 1 = unacceptable, 2 = poor, 3 = acceptable, 4 = good, 5 = excellent. Artifact severity and reader preference between sequences were also graded on a separate five-point scale: 1 = standard better, 2 = standard slightly better, 3 = equal, 4 = CS slightly better, 5 = CS better.

### 2.5. Statistical Analysis

All data were analyzed using the software package MedCalc Statistical Software version 19.3.1 (MedCalc Software Ltd., Ostend, Belgium). We determined the differences in volume measurement between the two sequences by obtaining the original volume for each region. The principal analyses focused on global brain volumetric measurements, whereas regional volumetric analyses were performed to evaluate agreement across individual brain structures. We conducted a paired t-test because the volume difference between the sequences for the entire dataset was deemed more important than the volume difference per patient [12]. We also evaluated the mean difference between CS-MPRAGE and standard MPRAGE-derived volume measures of each brain location using Bland–Altman plots. The qualitative and quantitative imaging parameters were also compared. Inter-method reliability was measured with the Pearson correlation coefficient (r) and the intraclass correlation coefficient (ICC). ICC was calculated using a two-way model with absolute agreement based on single measurements. ICC values were interpreted as follows: <0.50 = poor, 0.50–0.75 = moderate, 0.75–0.90 = moderate, >0.9 = excellent reliability [16].

## 3. Results

### 3.1. Study Cohort

Seven patients were excluded due to data errors (*n* = 3), structural abnormalities (*n* = 2), or severe artifacts (*n* = 2) (Figure 1). The final cohort comprised 40 patients (7 men, 33 women; mean age, 27.0 years; range, 19–40 years). MRI indications included headache (*n* = 23), dizziness (*n* = 10), loss of consciousness (*n* = 3), and other nonspecific symptoms (*n* = 4).

### 3.2. Acquisition Time

The mean acquisition time was 1 min 46 s for CS–MPRAGE and 5 min 20 s for standard MPRAGE, representing a significant reduction in scan duration (*p* < 0.001).

### 3.3. Quantitative Analysis

Whole-brain volume measurements were similar between standard-MPRAGE and CS-MPRAGE (1176.89 ± 105.36 vs. 1181.14 ± 105.62 mL, *p* = 0.27) with excellent reliability (r = 0.988; ICC 0.9754–0.9931). Gray matter and regional lobe volumes, except for the occipital lobe, showed no statistically significant differences between sequences.

**Figure 2:**
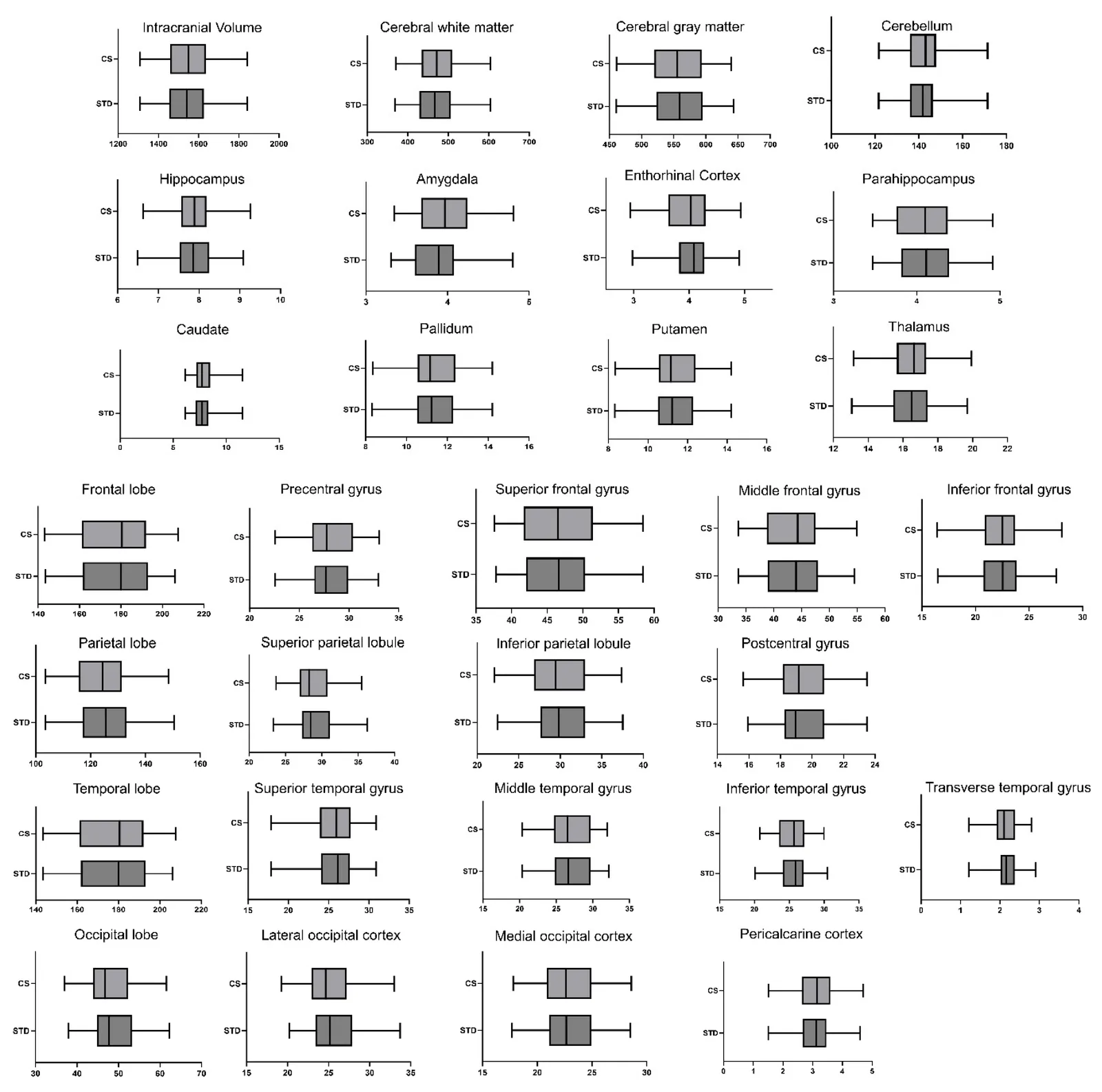
Comparison of standard MPRAGE and CS-MPRAGE; volume measurement; CS, Compressed sensing MPRAGE; STD, standard MPRAGE. Volume measurements showed similar results from the two sequences.

**Figure 3:**
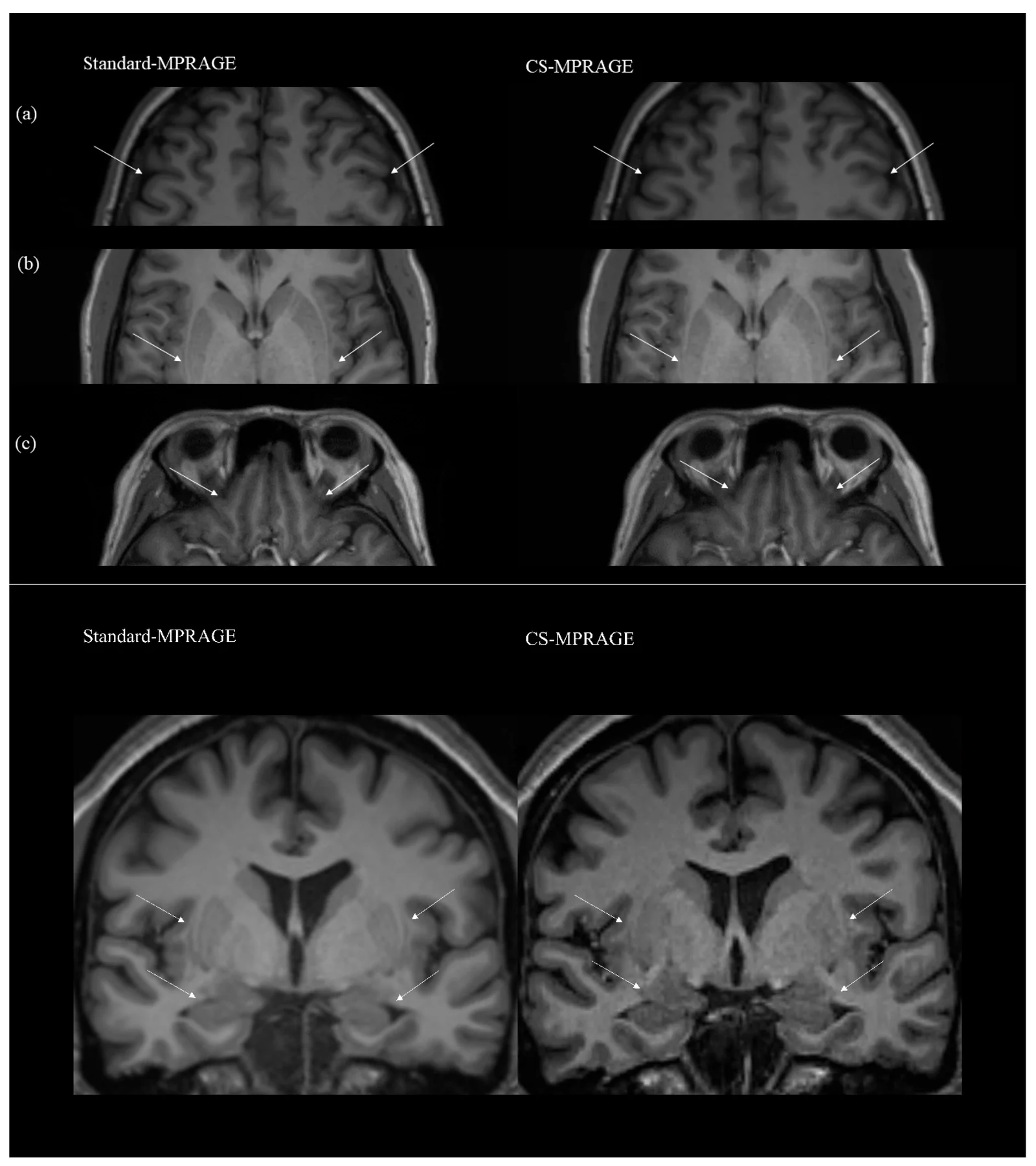
Comparison of standard MPRAGE and CS-MPRAGE top Axial CS-MPRAGE shows more blurring in the frontal cortex of the highest convexity (**a**) and basal area (**c**) and an indistinct margin of the basal ganglia (**b**) than standard MPRAGE, as indicated by the arrows. bottom Coronal CS-MPRAGE shows degraded image quality with blurred borders in medial temporal structures and basal ganglia, as indicated by the arrows.

White matter volume measured on the standard MPRAGE was significantly smaller than that measured on CS-MPRAGE (469.15 ± 55.74 vs. 473.32 ± 55.70 mL, *p* = 0.02). The CS-MPRAGE also yields slightly larger volumes for deep gray matter, hippocampus, and amygdala (Figure 2, Table 2). The blurring of tissue interfaces on CS-MPRAGE resulted in minor overestimation of the volume of white matter and basal ganglia (Figure 3).

**Figure 4:**
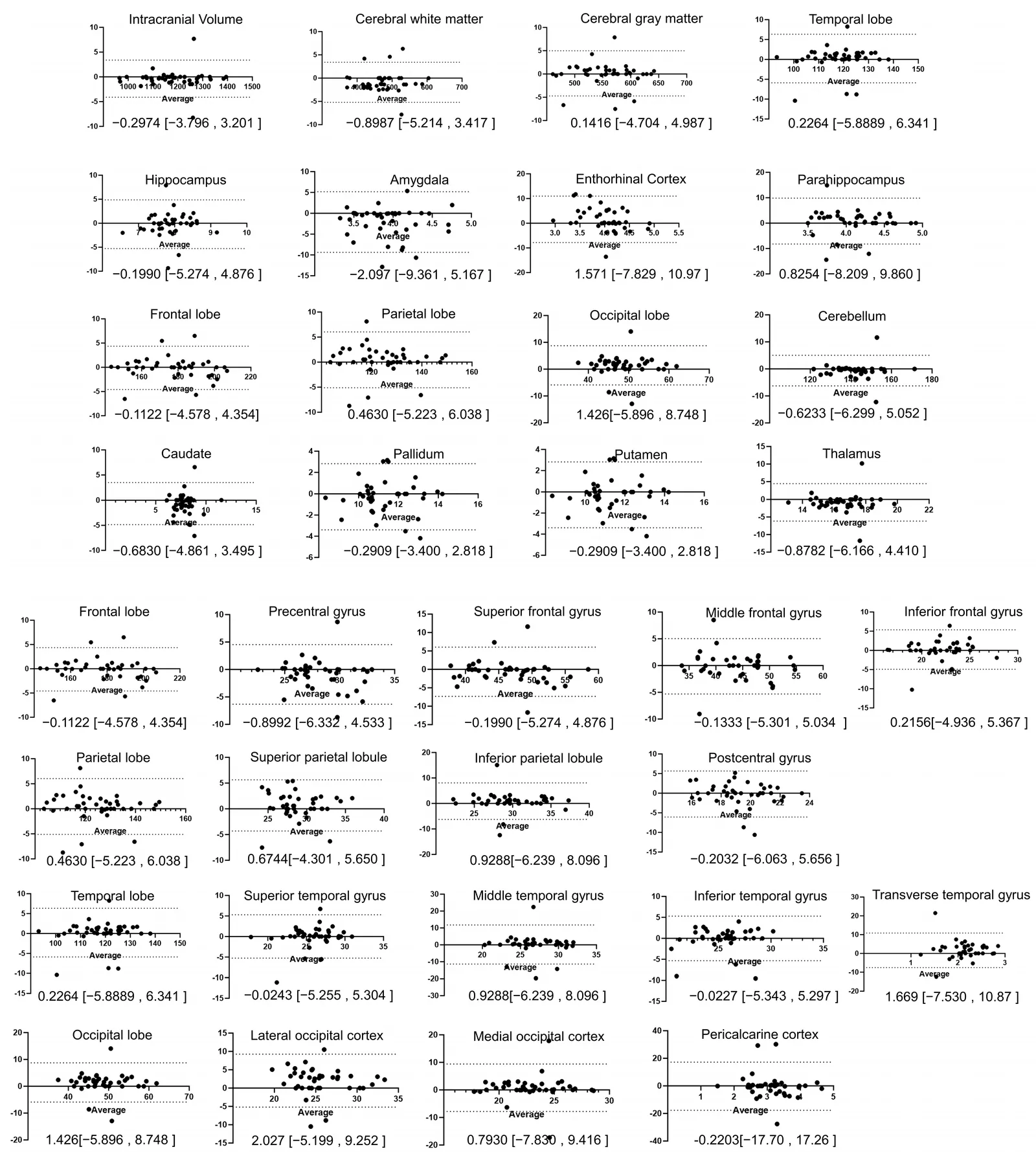
Comparison of standard MPRAGE and CS-MPRAGE; Bland–Altman plot; CS, Compressed sensing MPRAGE; STD, standard MPRAGE Bland–Altman analysis showed good agreement between the volumetric results from the two sequences.

Bland–Altman analysis showed good agreement between the volumetric results from the two sequences. Mean differences were negative for cerebral white matter, hippocampus and amygdala, as well as across the basal ganglia structures (Figure 4, Supplementary Table S1).

The CNR of gray–white matter was significantly higher for CS-MPRAGE compared to standard MPRAGE (left; 30.45 ± 11.21 vs. 49.53 ± 32.71, right; 30.73 ± 11.97 vs. 46.89 ± 30.40, *p* = 0.0001). In contrast, the SNR in the centrum semiovale, putamen, cerebellum and pons showed no significant difference between sequences (Table 3).

### 3.4. Qualitative Analysis

The observers reported the overall image quality of standard MPRAGE was significantly better than that of CS-MPRAGE (4.94 ± 0.24 vs. 4.54 ± 0.55, *p* < 0.0001). The same pattern was observed for gray–white matter differentiation and deep gray matter delineation. Artifacts were more frequent with CS-MPRAGE (41.25%, *n* = 33) than with standard MPRAGE (5.00%, *n* = 4; *p* < 0.0001) (Table 4). When analyzing the specific cause of the artifact, CS-MPRAGE exhibited significantly more blurring, especially in the central area. Regarding reader preference, observers rated the two sequences as equal in 65.0% of cases (52 of 80 observations), preferred standard MPRAGE in 33.75% (27 of 80 observations), and selected CS–MPRAGE in only a small minority of evaluations.

## 4. Discussion

In this study, we compared brain volume measurements using standard MPRAGE and CS accelerated MPRAGE. The present study was designed as a technical validation to determine whether CS-MPRAGE provides volumetric measurements comparable to those obtained with standard MPRAGE under controlled conditions. Therefore, we intentionally enrolled relatively young participants without structural brain abnormalities to minimize potential confounding effects of age-related atrophy or neurodegenerative changes. With CS, we achieved reliable whole-brain MPRAGE images suitable for volumetric measurement while reducing the scan time to approximately one-third of that required for standard MPRAGE. Although standard MPRAGE received slightly higher overall image quality scores and CS-MPRAGE exhibited a higher artifact rate, these differences did not adversely affect volumetric measurement, indicating that CS-MPRAGE remains suitable for quantitative brain volumetry. Our study found that CS-MPRAGE performs comparably to standard MPRAGE in measuring the volumes of large brain structures, such as the entire cortex. However, volumetric measurements of smaller structures in the medial temporal region, such as the entorhinal cortex and amygdala, differed between CS-MPRAGE and standard MPRAGE. These regions are known to be challenging to segment compared with other structures, such as the caudate nucleus, owing to their anatomical complexity, small size, and susceptibility to atrophic changes [17,18].

A well-known drawback of the CS technique is image blurring, particularly at the gray–white matter border, which can result from high acceleration factors, random undersampling of k-space in the peripheral location, or iterative reconstruction [19,20,21]. Blurring at this interface could have affected volumetric measurements. For example, white matter volume with CS-MPRAGE was greater than that obtained with standard MPRAGE, whereas gray matter volume showed the opposite trend, although the differences were not statistically significant. We performed volume measurement using Neurophet AQUA, which calculates surface-based approach rather than simple voxel count [12,22,23]. Because surface reconstruction is highly dependent on the image contrast, differences in gray–white matter contrast and the surrounding structures may introduce variability in segmentation, particularly in small and anatomically complex areas, potentially leading to inconsistency in results. Previous studies have reported inconsistent volume measures across the frontal and temporal regions because of the susceptibility effects near the air-bone interface, including paranasal sinuses or mastoid air cells [24]. We observed similar volumetric results with both MPRAGE and CS-MPRAGE in the inferior temporal cortex and frontal pole. We assume those susceptibility effects may have similarly contributed to the image distortion in both sequences.

One of the greatest advantages of incorporating CS into MPRAGE is the substantial reduction in scan time, which can improve patient comfort and compliance [25]. Longer scan times increase the likelihood of motion-related image degradation. Previous research has reported that up to 29% of inpatient or emergency department examinations and 7% of outpatient studies are affected by motion, often requiring repeat scans that increase financial burden and workload [26,27]. Our findings indicate that CS-MPRAGE provides a reliable acquisition technique suitable for daily clinical practice, producing consistent volumetric results while reducing scan time to approximately one-third of that required for standard MPRAGE. In many neurodegenerative disorders, progressive neural atrophy can be quantified using MRI-based volumetric measurements. In clinical practice, these measurements can provide insights into disease progression, the risks of rapid clinical deterioration, therapeutic effects and prognosis. Although our study results indicated a higher frequency of artifacts with CS-MPRAGE, most were related to susceptibility effects rather than motion. These issues may potentially be mitigated by further optimization of sequence parameters. Implementing CS-accelerated MPRAGE in clinical practice could facilitate quantitative neuroimaging assessment while improving workflow efficiency in imaging facilities.

In this study, we included only relatively young participants (aged 19–40 years) without intracranial lesions to minimize the confounding effects of physiologic aging and brain degeneration. It is well established that the brain undergoes degenerative changes with aging, including volume loss, particularly in the frontal and temporal cortices. Although brain atrophy is influenced by multiple factors, including environmental and genetic factors, and biological aging does not fully represent all degenerative processes, studies have reported that brain volume decreases by approximately 5% per decade after the age of 40 on average [11,28]. Because there is currently no defined gold-standard non-imaging method for estimating brain volume [29], we did not include age or physiologic degeneration as a covariate in the present analysis.

On visual inspection, CS-MPRAGE images exhibited more noise and artifacts than standard MPRAGE, particularly in peripheral brain regions. This finding was expected, and the mean difference in visual rating scores between the two sequences was small. These results suggest that CS-MPRAGE could potentially serve as an alternative to standard MPRAGE in clinical brain imaging protocols, enabling more efficient use of MRI resources. However, additional validation studies are needed to confirm the comparability of CS-MPRAGE with standard MPRAGE in clinical settings, particularly in patients with various neurodegenerative disorders.

Bland–Altman analysis demonstrated minimal systematic bias between CS-MPRAGE and standard MPRAGE for most brain structures. Slight negative mean differences were observed in cerebral white matter, hippocampus, amygdala, and several basal ganglia structures, indicating that standard MPRAGE yielded marginally lower volumetric measurements than CS-MPRAGE. Differences in contrast between gray matter and adjacent structures may contribute to segmentation variability. However, the average differences were close to zero, indicating two measurement methods produced highly consistent results. Although statistically significant differences were observed in several brain locations, including amygdala, entorhinal cortex, and occipital lobe, the absolute mean differences were small. Furthermore, Bland–Altman analysis demonstrated minimal systematic bias and relatively narrow 95% limits of agreement across most brain regions, indicating that inter-sequence variability was limited. Taken together, these findings suggest that the observed differences are unlikely to materially affect clinical interpretation or longitudinal volumetric assessment, supporting the use of CS-MPRAGE as a reliable alternative to standard MPRAGE for automated brain volumetry.

This study has several limitations. First, selection bias could exist because this study was conducted at a single referral center with a relatively small sample size. We intentionally included only images without morphological alteration to facilitate the comparison of absolute values. Further studies with larger sample sizes will be valuable for validation. Second, we intentionally included relatively young subjects without structural brain abnormalities to minimize the potential confounding effects of age-related atrophy and pathological changes when comparing volumetric measurements between imaging sequences. We assumed that the cohort’s brains were not yet affected by degenerative changes and regarded all subjects as representing the normal range of brain volume based on their age and MRI morphology. Accordingly, the generalizability of our findings to elderly individuals and patients with neurodegenerative diseases remains to be established. Third, although the neuroradiologists were blinded to pulse sequences during evaluation, they might have recognized the imaging characteristics specific to each pulse sequence, potentially introducing observer bias. Moreover, the acquisition order could have affected image quality, as later acquisitions may have been more susceptible to motion artifacts. We tried to minimize bias by (1) randomizing the acquisition order and (2) performing both qualitative analyses and quantitative analyses. Finally, we assessed brain volume using a single commercially available automated segmentation software. Because standard MPRAGE is not a true reference standard for absolute brain volumetry, the present study evaluated the agreement of volumetric measurements between standard MPRAGE and CS-MPRAGE rather than the absolute accuracy of CS-MPRAGE. We found no significant differences in volumetric measurements between the two sequences. Further research using multiple segmentation tools and independent reference standards could provide additional insights into the accuracy and robustness of volumetric measurements. In addition, volume measurements of small medial temporal structures showed inconsistent results, and visual confirmation of these small brain regions remains challenging. Improving the consistency of these assessments will require further methodological refinement and validation.

## 5. Conclusions

In conclusion, CS-MPRAGE provides high-quality three-dimensional images and reliable volumetric data in significantly less acquisition time than standard MPRAGE. In this technical validation study of healthy young adults, CS-MPRAGE demonstrated volumetric measurements comparable to those obtained with standard MPRAGE while substantially reducing scan time. These findings support the feasibility of CS-MPRAGE as a time-efficient alternative for brain volumetric imaging, although further validation in elderly individuals with neurodegenerative changes is required before broader clinical application.

## Figures and Tables

**Table 1:** Imaging parameters.

	Standard MPRAGE	CS MPRAGE
TR/TE (ms)	2300/2.98	2300/2.9
Echo Spacing (ms)	7.14	7.86
Inversion time (ms)	900	900
Flip angle (°)	9	9
Acceleration Method	GRAPPA	Compressed sensing
Acceleration factor	2	5
Slice thickness (mm)	1.0	1.0
Number of slices	176	176
Acquisition time	5′20″	1′46″

MPRAGE, Magnetization Prepared Rapid Acquisition Gradient Echo; CS, compressed sensing; TR, repetition time; TE, echo time.

**Table 2:** Volume measurement using standard MPRAGE and CS-MPRAGE.

		Standard MPRAGE (Mean ± SD)	CS-MPRAGE (Mean ± SD)	*p*	ICC
**Intracranial Volume**		1546.63 ± 132.97	1551.21 ± 132.88	0.32	0.988
**Whole-brain**		1176.89 ± 105.36	1181.14 ±105.62	0.27	0.987
	Gray matter	557.12 ± 45.59	556.34 ±47.84	0.72	0.989
	White matter	469.15 ± 55.74	473.32 ±55.70	0.02	0.990
	Frontal lobe	177.30 ± 17.49	177.53 ± 17.94	0.72	0.987
	Precentral gyrus	28.11 ± 2.48	28.37 ± 2.63	0.70	0.939
	Superior frontal gyrus	46.50 ± 5.12	46.82 ± 5.43	0.72	0.975
	Middle frontal gyrus	43.89 ± 5.54	43.97 ± 5.68	0.88	0.990
	Inferior frontal gyrus	22.19 ± 2.48	22.13 ± 2.42	0.88	0.987
	Frontal pole	1.98 ± 0.26	1.92 ± 0.28	0.37	0.929
	Parietal lobe	124.73 ± 11.90	124.17 ± 12.09	0.31	0.979
	Superior parietal lobule	29.06 ± 2.83	28.87 ± 2.85	0.97	0.984
	Inferior parietal lobule	29.96 ± 3.65	29.67 ± 3.66	0.74	0.979
	Postcentral gyrus	19.24 ± 1.83	19.29 ± 3.67	0.91	0.952
	Temporal lobe	118.55 ± 10.19	118.25 ± 9.78	0.57	0.966
	Superior temporal gyrus	25.78 ± 2.77	25.76 ± 2.64	0.75	0.985
	Middle temporal gyrus	26.89 ± 3.04	26.84 ± 3.09	0.92	0.928
	Inferior temporal gyrus	25.52 ± 2.32	25.52 ± 2.25	0.84	0.977
	Transverse temporal gyrus	2.15 ± 0.37	2.11 ± 0.37	0.67	0.988
	Occipital lobe	48.83 ± 5.56	48.14 ± 5.51	0.02 *	0.972
	Lateral occipital cortex	25.79 ± 3.17	25.28 ± 3.19	0.97	0.979
	Medial occipital cortex	23.04 ± 2.75	22.86 ± 2.74	0.97	0.961
	Pericalcarine cortex	3.12 ± 0.63	3.14 ± 0.66	0.72	0.952
	Cerebellum	141.99 ± 10.13	142.88 ± 10.15	0.21	0.951
**Deep gray matter**					
	Caudate	7.83 ± 0.97	7.89 ± 0.97	0.07	0.992
	Pallidum	4.61 ± 0.60	4.58 ± 0.58	0.42	0.954
	Putamen	11.34 ± 1.31	11.37 ± 1.32	0.26	0.995
	Thalamus	16.47 ± 1.37	16.61 ± 1.40	0.06	0.970
**Medial temporal lobe**					
	Hippocampus	7.87 ± 0.51	7.89 ± 0.50	0.65	0.958
	Amygdala	3.90 ± 0.36	3.98 ± 0.37	0.001 *	0.958
	Enthorhinal cortex	4.03 ± 0.39	3.98 ± 0.45	0.006 *	0.949
	Parahippocampus	4.13 ± 0.38	4.09 ± 0.38	0.25	0.941

MPRAGE, Magnetization Prepared Rapid Acquisition Gradient Echo; CS, compressed sensing; ICC, intraclass correlation coefficient; * statistically significant.

**Table 3:** Comparison of CNR and SNR.

		Standard MPRAGE (Mean ± SD)	CS-MPRAGE (Mean ± SD)	*p*
**CNR**				
	Left	30.45 ± 11.21	49.53 ± 32.71	0.0001
	Right	30.73 ± 11.97	46.89 ± 30.40	0.0001
**SNR**				
	Left Centrum Semiovale	47.18 ± 12.24	50.95 ± 16.72	0.1834
	Right Centrum Semiovale	46.83 ± 12.39	50.32 ± 14.77	0.4772
	Left Putamen	32.22 ± 8.72	29.65 ± 10.16	0.1916
	Right Putamen	31.12 ± 9.62	28.44 ± 8.10	0.0707
	Left Cerebellum	36.41 ± 12.20	33.67 ± 10.03	0.1864
	Right Cerebellum	37.81 ± 11.24	34.83 ± 11.69	0.1946
	Pons	25.55 ± 7.03	25.8 ± 8.94	0.7699

MPRAGE, Magnetization Prepared Rapid Acquisition Gradient Echo; CS, compressed sensing.

**Table 4:** Comparison of qualitative analysis between standard MPRAGE and CS-MPRAGE.

	Standard MPRAGE	CS-MPRAGE	*p* Value
Overall image quality	4.94 ± 0.24	4.54 ± 0.55	<0.0001
GW differentiation	5.00 ± 0.00	4.91 ± 0.40	0.05
DG differentiation	5.00 ± 0.00	4.71 ± 0.51	<0.0001
Artifact	5.00% (*n* = 4/80)	41.25% (*n* = 33/80)	<0.0001

Data are mean ± standard deviation and averaged scores of two observers. MPRAGE, Magnetization Prepared Rapid Acquisition Gradient Echo; CS, Compressed sensing; GW, gray–white matter; DG, deep gray matter.

## Data Availability

The datasets used and/or analyzed during the current study available from the corresponding author on reasonable request, but there could be certain process to release the data due to the local policy.

## Supplementary Materials

The following supporting information can be downloaded at: https://www.mdpi.com/article/10.3390/tomography12090121/s1, Figure S1. Brain segmentation using Neurophet AQUA. Table S1. Comparison of standard MPRAGE and CS-MPRAGE; Bland–Altman analysis.

## Abbreviations

The following abbreviations are used in this manuscript:

CS	Compressed Sensing
MPRAGE	Magnetization Prepared Rapid Acquisition Gradient Echo
MRI	Magnetic resonance imaging
