# Supplementary material for: Validation of Compressed Sensing Accelerated Magnetization Prepared Rapid Acquisition Gradient Echo for Fast and Reliable Brain Volume Measurement
Source: Tomography. 2026 Aug 24;12(9):121. doi: 10.3390/tomography12090121 (PMC13611507; doi:10.3390/tomography12090121)
Supplement: Supplementary file 1 [file tomography-12-00121-s001.zip › tomography-4445997-supplementary.pdf]

Supplementary Materials

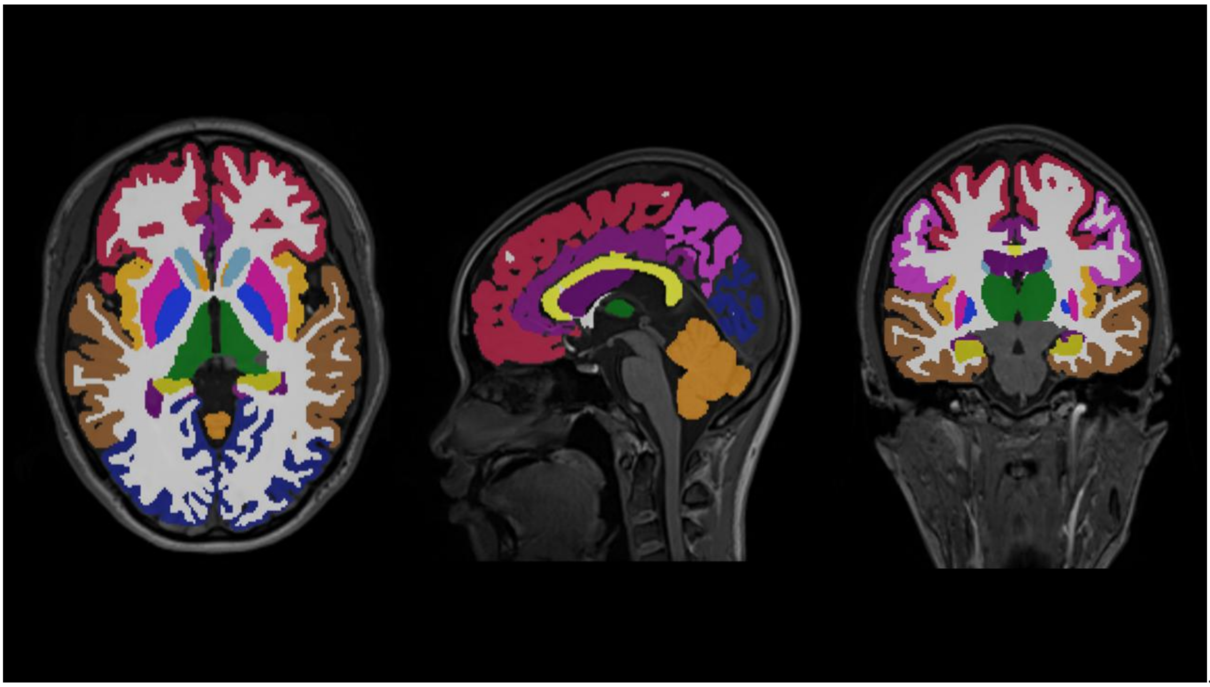

Figure S1. brain segmentation using Neurophet AQUA.

Table S1. **Comparison of standard MPRAGE and CS-MPRAGE;** Bland−Altman analysis

	Mean bias	95% limits of agreement
**Intracranial Volume**	-0.2974	-3.796 to 3.201
**Whole brain**	-0.3616	-4.113 to 3.390
Gray matter	0.1416	-4.704 to 4.987
White matter	-0.8987	-5.214 to 3.417
Frontal lobe	-0.1122	-4.578 to 4.354
Precentral gyrus	-0.8992	-6.332 to 4.533
Superior frontal gyrus	-0.1990	-5.274 to 4.876
Middle frontal gyrus	-0.1333	-5.301 to 5.034
Inferior frontal gyrus	0.2156	-4.936 to 5.367
Parietal lobe	0.4630	-5.223 to 6.038
Superior parietal lobule	0.6744	-4.301 to 5.650
Inferior parietal lobule	0.9288	-6.239 to 8.096
Postcentral gyrus	-0.2032	-6.063 to 5.656
Temporal lobe	0.2264	-5.889 to 6.341
Superior temporal gyrus	-0.0243	-5.255 to 5.304
Middle temporal gyrus	0.9288	-6.239 to 8.096
Inferior temporal gyrus	-0.0227	-5.343 to 5.297
Transverse temporal gyrus	1.669	-7.530 to 10.870
Occipital lobe	1.426	-5.896 to 8.748
Lateral occipital cortex	2.027	-5.199 to 9.252
Medial occipital cortex	0.7930	-7.830 to 9.416
Pericalcarine cortex	-0.2203	-17.70 to 17.26
Cerebellum	-0.6233	-6.299 to 5.052
**Deep gray matter**		

Caudate	-0.6830	-4.861 to 3.495
Pallidum	-0.2909	-3.400 to 2.818
Putamen	-0.2909	-3.400 to 2.818
Thalamus	-0.8782	-6.166 to 4.410
**Medial temporal lobe**		
Hippocampus	-0.1990	-5.274 to 4.876
Amygdala	-2.097	-9.361 to 5.167
Enthorhinal cortex	1.571	-7.829 to 10.970
Parahippocampus	0.8254	-8.209 to 9.860
